# Molecular Evolutionary Growth of Ultralong Semiconducting Double‐Walled Carbon Nanotubes

**DOI:** 10.1002/advs.202205025

**Published:** 2022-11-24

**Authors:** Jun Gao, Yaxin Jiang, Sibo Chen, Hongjie Yue, He Ren, Zhenxing Zhu, Fei Wei

**Affiliations:** ^1^ Beijing Key Laboratory of Green Chemical Reaction Engineering and Technology Department of Chemical Engineering Tsinghua University Beijing 100084 China

**Keywords:** carbon nanotubes, growth mechanism, molecular evolutionary, template auto‐catalysis

## Abstract

The self‐assembling preparation accompanied with template auto‐catalysis loop and the ability to gather energy, induces the appearance of chirality and entropy reduction in biotic systems. However, an abiotic system with biotic characteristics is of great significance but still missing. Here, it is demonstrated that the molecular evolution is characteristic of ultralong carbon nanotube preparation, revealing the advantage of chiral assembly through template auto‐catalysis growth, stepwise‐enriched chirality distribution with decreasing entropy, and environmental effects on the evolutionary growth. Specifically, the defective and metallic nanotubes perform inferiority to semiconducting counterparts, among of which the ones with double walls and specific chirality (*n*, *m*) are more predominant due to molecular coevolution. An explicit evolutionary trend for tailoring certain layer chirality is presented toward perfect near‐(2*n*, *n*)‐containing semiconducting double‐walled nanotubes. These findings extend our conceptual understanding for the template auto‐catalysis assembly of abiotic carbon nanotubes, and provide an inspiration for preparing chiral materials with kinetic stability by evolutionary growth.

## Graphical abstract

An abiotic system with evolutionary characteristics is extraordinary. Herein, the molecular evolution in the growth of ultralong carbon nanotubes is demonstrated. Specific CNTs are revealed to be extremely distinctive in the evolutionary sense, though merely owning delicate structural difference with others, especially, an explicit trend toward perfect near‐(2*n*, *n*)‐containing semiconducting double‐walled nanotubes indicates the effect of coevolution.

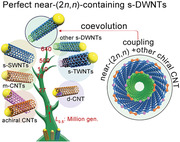

## 1 Introduction

Self‐assembly based on specific template is universal in the biotic world, stretching over multiple scales and even synthesizing chiral structures from achiral building blocks.^[^ ^1^ ^]^ Template self‐assembly is closely associated with the origin of life and biomolecular evolution,^[^ ^2^, ^3^, ^4^, ^5^, ^6^, ^7^, ^8^, ^9^ ^]^ which rely on the interaction between energy gathering ability and template replication.^[^ ^6^ ^]^ The template replication is generally conducted in an auto‐catalysis way. Nevertheless, the small‐molecule auto‐catalysis and the reflexively auto‐catalytic sets of template polymers are crucially different. The former requires various kinds of chemical steps, whereas the template polymers can be synthesized through simple iterations or a small set of canonical reactions, thus their auto‐catalysis exhibit more targeted and specific behaviors,^[^ ^6^ ^]^ yielding the unique regime called template auto‐catalysis (TAC). In the TAC process with the ability of energy gathering, the kinetic competitions among different pathways are decisive for the final product distribution, while the competition criterion is kinetic stability, which rationally leads to the preference on chiral assemblies against achiral ones.^[^ ^4^ ^]^ The iterations of a characteristic auto‐catalysis loop provide positive feedback, making the assemblies with greater kinetic stability more abundant and driving the evolution of inanimate matter.

As one kind of pure‐carbon material with sp^2^ nanostructure, defect‐free carbon nanotubes (CNTs) with a macro length possess unique atomic structure and excellent properties for high‐end applications.^[^ ^10^, ^11^, ^12^, ^13^, ^14^ ^]^ To prepare such ultralong perfect CNTs, chemical vapor deposition (CVD) growth is the most promising approach,^[^ ^15^ ^]^ which involves the nucleation of the nascent seeds and subsequent elongation through atomic self‐assembly. It has been demonstrated that the long‐range assembly of carbon atoms follows the vapor‐liquid‐solid mode^[^ ^16^ ^]^ dominated by the kinetic elongation process. Such a growth regime contributes to defect‐free ultralong CNTs with an ultrafast growth rate over 80 µm s^−1^.^[^ ^17^, ^18^ ^]^ It is equivalent to the turnover frequency ≈10^6^ s^−1^,^[^ ^18^, ^19^ ^]^ indicating that the assembly rate of carbon atoms onto the circumferential template surpasses almost all the industrial catalytic reactions. But actually, in the catalytic CVD process, graphitic carbon encapsulating the catalyst is more energetically favorable than CNT,^[^ ^20^ ^]^ whose growth is out of thermal equilibrium and needs continuous energy input.^[^ ^21^ ^]^ For such a self‐assembly process dominated by kinetics and far from equilibrium, the indispensable setups for evolutionary growth are intrinsic.

Furthermore, during the elongated growth, the diversiform CNT edges provide different templates and assembling pathways, and the growth actually means innumerable assembling iterations according to the configurational information embedded within the template. In some special situations like cloning growth, where open‐end CNTs serve as the seeds for epitaxial growth,^[^ ^22^, ^23^, ^24^ ^]^ the nucleation process can be circumvented and metal catalysts are not prerequisites, actually verifying the auto‐catalysis ability of CNT. Chiral CNTs showed obvious superiority over achiral (armchair and zigzag) CNTs in cloning growth,^[^ ^24^ ^]^ also demonstrating the significance of chiral assembling forms for CNTs. Each round of circumferential atomic assembly during the CNT growth can be regarded as the production of a new “generation,” similar to the transmission of hereditary information via gene replication in the bio‐systems. The kinetic competitions among different TAC pathways further provide a basis for evolutionary growth of ultralong CNTs, with assembling iterations over hundreds of millions of generations.

Herein, we will demonstrate the molecular evolutionary growth of ultralong CNTs determined by TAC mechanism. First, TAC loops drive achiral CNTs into extinction while chiral CNTs behave as template replicators. Thus, under the competition among different assembling pathways, the distributions of chiral CNTs gradually become narrower as length increases. Specific CNT species are revealed to be extremely distinctive in the evolutionary sense, though merely owning delicate structural difference with others. Especially, by regulating the environment, an explicit trend toward near‐(2*n*, *n*)‐containing semiconducting double‐walled CNTs (s‐DWNTs) was shown after sorting out the predominant species. Such molecular evolutionary growth has provided a strategy for tailoring local chirality of a coupled system, which contributes to preparing chiral materials with topologically complex atomic structure and kinetic stability.

## 2 Results and Discussion

### 2.1 Bandgap Determines the Primary Evolution

**Figure 1:**
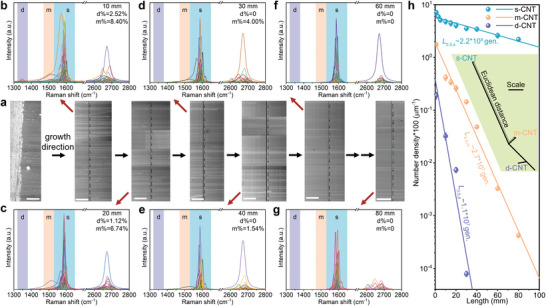
Gradual evolution toward defect‐free ultralong semiconducting CNTs. a) Typical scanning electron microscope (SEM) images of catalyst regions and multiple length positions to demonstrate the morphology and density of CNTs along the growth direction. The marks on the substrates are fabricated to expediently locate CNTs and conduct characterizations. Scale bars, 400 µm. b–g) The Raman spectra at multiple positions with the length of 10 mm (b), 20 mm (c), 30 mm (d), 40 mm (e), 60 mm (f), and 80 mm (g) to identify the content of defective CNTs, metallic CNTs and semiconducting CNTs, marked by “d”, “m”, and “s”, respectively. h) Number density of d‐, m‐, and s‐CNTs along the length, showing extremely different decaying rates. The *L*_0.5_ represents the half‐length depicted with the number of generations, noted as “gen.”. Inset: phylogenetic tree of d‐, m‐, and s‐CNT based on Euclidean distances calculated from the number density at different positions. The line with arrows ending marks the Euclidean distance between s‐CNT and m‐CNT, as an example.

The CNTs can be classified as metallic CNTs (m‐CNTs), semiconducting CNTs (s‐CNTs) and defective CNTs (d‐CNTs), which are mainly distinguished through Raman spectra. The d‐CNTs indicate the CNTs showing measurable d band signal in Raman spectra (with a D/G intensity ratio larger than 10^−2^). Then the s‐CNTs and m‐CNTs are differentiated by the line‐shapes of G band. It is a well‐acknowledged^[^ ^18^, ^25^, ^26^ ^]^ and reliable method, as we eliminated the interference from air^[^ ^27^ ^]^ in the characterizations, and the as‐prepared few‐walled CNTs were resonant with the laser wavelength we used, due to the narrow distribution of outer diameter (seen in the Supporting Information) and the quantum coupling between layers.^[^ ^28^ ^]^ During the CNT growth, the number of total CNTs decreases as the length increases, as shown in **Figure** **1**a. Furthermore, the number of defective CNTs (d‐CNTs), metallic CNTs (m‐CNTs), and semiconducting CNTs (s‐CNTs) all exponentially decay with length, but with extremely different decaying rates. Raman spectra at multiple length positions explicitly perform gradual enrichment toward defect‐free s‐CNTs (Figure 1b–g), manifesting that the evolutionary growth becomes prominent as the increase of growth duration. The results at the length longer than 60 mm (Figure 1f,g) behave none D‐band (1350 cm^−1^) or metallic‐G‐band (1580 cm^−1^) characteristics, revealing an ultrahigh purity of defect‐free s‐CNT. More evidence verifying the perfect structure of as‐prepared CNTs can be seen in Figures S1–S4 and Section S1 in the Supporting Information.

To describe the decaying rate, we properly introduce the half‐length *L*_0.5_ to depict the required number of atomic assembly generations when the number density of CNTs decreases by half compared to the initial value (Section S2 and Figure S5, Supporting Information). The growth witnesses that d‐CNTs decay primarily, much more rapidly than m‐CNTs and s‐CNTs, while the decaying rate for m‐CNTs is ≈8 times faster than s‐CNTs, with a comparison of *L*_0.5_ between 2.7 × 10^7^ generations for m‐CNTs and 2.2 × 10^8^ generations for s‐CNTs (Figure 1h). Moreover, Euclidean distance (Section S3, Supporting Information), a widely‐used parameter for characterizing species or populations in biology and ecology,^[^ ^29^, ^30^ ^]^ was used to quantitatively measure the evolutionary divergence among the three kinds of CNTs (inset of Figure 1h). The Euclidean distance from perfect s‐CNT to d‐CNT and perfect m‐CNT are both over two times longer than that between the latter two (Tables S1 and S2, Supporting Information), which means that s‐CNT, the intrinsic superior species, is sufficiently far from m‐CNT and d‐CNT in the evolutionary dimension.

The results indicate it is the bandgap, which describes the functional difference, rather than the topological atomic structure, that determines the evolutionary divergence of populations, also consistent with the regimes in biotic world.^[^ ^31^ ^]^ The s‐CNTs and m‐CNTs with adjacent chiral indices merely behave tiny difference in atomic structure. But the s‐CNTs, especially those within specific bandgap range, possess the rate of template assembly about an order of magnitude larger than m‐CNTs,^[^ ^19^ ^]^ which endows specific s‐CNTs with greater kinetic stability and superiority in evolutionary growth. Whereas, the defects formed in the assembly process would destroy the sp^2^ atomic structure and resultant bandgap structure, as well as the strongly‐coupled relation between the template assembly and bandgap. Thus, d‐CNTs become markedly inferior in the perspectives of kinetic stability and evolution. The bandgap‐coupled TAC kinetics result in the obvious divergence of Euclidean distance and the bio‐like evolution toward defect‐free ultralong semiconducting CNTs.

### 2.2 Superiority of Double‐Walled s‐CNTs in Evolutionary Growth

**Figure 2:**
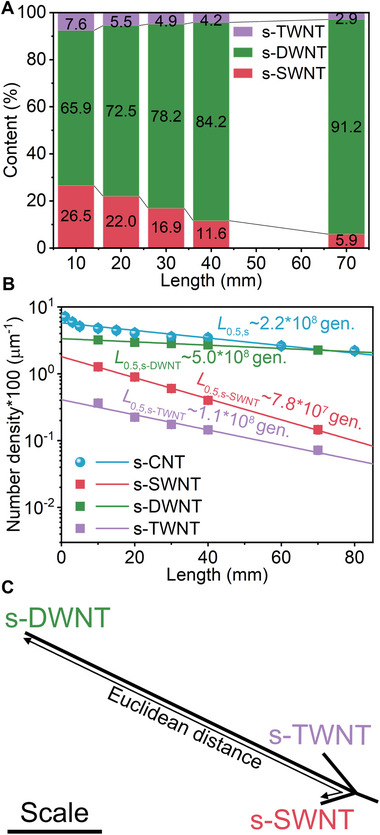
The evolution within the s‐CNTs caused by different wall numbers. a) The variation of the s‐CNT content with different wall numbers as length increases. b) Comparison of the decaying rate among s‐CNT entirety and the portions of s‐SWNTs, s‐DWNTs, and s‐TWNTs. c) Phylogenetic tree of s‐SWNT, s‐DWNT, and s‐TWNT based on the calculated Euclidean distances. The line with arrows ending marks the Euclidean distance between s‐DWNT and s‐SWNT, as an example.

In evolutionary growth, s‐CNTs are the preferred populations compared with d‐CNTs and m‐CNTs. Actually, within the s‐CNT populations, some species are superior to others. The Raman spectra of as‐prepared CNTs, especially their RBM mode (**Figure** **2**a; Figure S6, Supporting Information), further reveal the evolution process of s‐CNTs with different walls. Semiconducting single‐walled carbon nanotubes (s‐SWNTs) and semiconducting triple‐walled carbon nanotubes (s‐TWNTs) decay more quickly while s‐DWNTs possess the longest half‐length *L*_0.5_ ≈5.0 × 10^8^ generations, as shown in Figure 2b. Further comparison of the half‐length *L*_0.5_ among these s‐CNTs demonstrate that, due to the existence of s‐SWNTs and s‐TWNTs, the decaying rate of s‐CNT entirety is much quicker than that of s‐DWNTs. Besides, the Euclidean distance from s‐DWNT to s‐TWNTs and s‐SWNTs are both over four times longer than that between the latter two (Tables S3 and S4, Supporting Information), revealing that s‐DWNTs are quite distinctive from s‐TWNTs and s‐SWNTs in the evolutionary sense (Figure 2c). It might be related to the unique conformation and layer‐to‐layer interactions of DWNTs, as the two layers share the same one catalyst and the growth behavior of one layer is markedly associated with the other. Due to the influence of mutual interaction and competition between layers, the coevolution of their template assembly exists. SWNTs are eliminated earlier due to the absence of coevolution and protection. Nevertheless, TWNTs are inferior as there are more possible combinations of inter‐wall interactions induced by complicated multibody effects, which would cause an adverse effect for coevolution.

### 2.3 Toward Perfect Near‐(2*n*, *n*)‐Containing Chiral Semiconducting DWNTs

**Figure 3:**
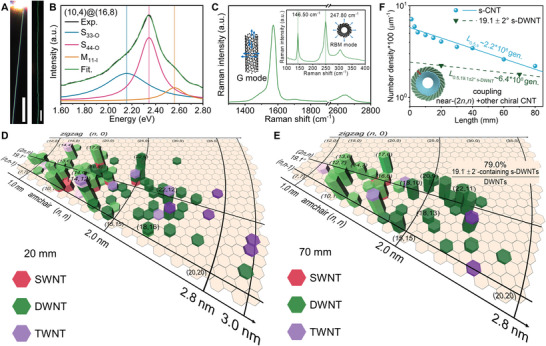
The chirality distribution and deep explorations for evolutionary features. a) Typical true‐color RRS images of an air‐suspended CNT (left) and a CNT on the substrate (right). The glaring light in the left image is the edge of the substrate under the illumination of laser. Scale bars, 100 µm. b) RRS spectra of a typical DWNT fitted by Lorentz peaks to identify the resonance energy. c) Raman spectra of the corresponding DWNT in (a). Inset is the radial breathing mode (RBM) range. (b) and (c) are combined to identify its chiral index to be (10, 4)@(16, 8). d,e) Chirality distribution of all CNT walls at the length positions of 20 mm (d) and 70 mm (e). The prismoids in the same colors but with different gradations represent the different walls of DWNTs and TWNTs. f) Comparison of the decaying rate between s‐CNT entirety and the s‐DWNTs with at least one wall in the 19.1 ± 2° range.

Further insights for the evolutionary growth were obtained by the exploration on the chirality distributions. Specifically, we analyzed the chirality distributions of as‐prepared CNTs with the assistance of Rayleigh resonance scattering (RRS) images and spectra, a convenient approach to characterize the chiral structure and indices of CNTs.^[^ ^32^, ^33^ ^]^ Both the CNTs on the substrate and the air‐suspended CNTs excluding the external interference behave homogeneous color in the RRS images, demonstrating the structural consistency of each ultralong CNT (**Figure** **3**a; Figures S1 and S2, Supporting Information). The consistent Rayleigh resonance peak values along an ultralong CNT also proved its perfect structure and the reliability of RRS spectra (Figure S3, Supporting Information). When combining RRS with Raman spectra, the chiral indices of different walls can be precisely assigned (Section S4, Supporting Information). Three representative samples were identified to be SWNT, DWNT, and TWNT, respectively (Figure 3b,c; Figure S7, Supporting Information). Furthermore, detailed chirality distributions of each wall from the CNTs at the length positions of 20 and 70 mm are presented in Figure 3d,e, Tables S5 and S6, and Data S1, Supporting Information.

The CNTs at 20‐mm‐length position spread over a relatively wide range of chirality (Figure 3d). The products are few‐walled CNTs, among of which DWNTs occupy 72.5%, while SWNTs and TWNTs cover 22% and 5.5%, respectively. It is noteworthy that some chiral species simultaneously exist in different types of wall layers, such as the (14,12) index, where four types of layers from DWNT and TWNT gather. It indicates that the chirality distribution at 20 mm is relatively scattered and the survival spaces for different wall layers overlap with each other. Obviously, the distribution of surviving CNTs at 70 mm behaves more concentrated, in the dimensions of wall number, chirality and diameter, which embodies the gradually‐decreased entropy due to continuous energy input under the TAC mechanism (Figure 3e). DWNTs dominate in total 68 CNTs with a proportion of 91.2%, and the chirality of all CNT walls is distributed around two discrete lines of (2*n*, *n*), i.e., 19.1° and (*n*, *n*−1). The preference on the line of 19.1° is obvious, especially for the DWNTs. The s‐DWNTs with at least one wall being in the 19.1 ± 2° range occupy a portion of 79.0% in all counted DWNTs. This is the first time to successfully regulate the chirality of a certain layer within incommensurate DWNTs. Meanwhile, a high semiconducting purity of 99.3% for all walls was achieved. It signifies that the molecular evolutionary growth is effective in tailoring chiral materials with customized topologically atomic structure in a non‐destructive way.

The explorations for the chirality distributions reveal the evolutionary characteristics of chiral angle within s‐DWNTs. Those s‐DWNTs with at least one wall being in the 19.1 ± 2° range behave an obviously slower decaying trend compared with their counterparts (Figure 3f), making them the dominant species in the evolutionary growth. Besides the aforementioned superiority of decaying rate, which is related to the lifetime, it should be noted that the intrinsically high growth rate might also be the case for their longer length, as many theoretical and experimental results have demonstrated the 19.1° SWNTs grown from solid catalysts possess the fastest growth rate due to their most edge dislocations.^[^ ^34^, ^35^, ^36^ ^]^ Moreover, our recent investigations demonstrated the markedly different growth rates between chiral s‐CNTs and chiral m‐CNTs, which originate from the bandgap‐coupled kinetics.^[^ ^18^, ^19^ ^]^ However, the relationship between the growth rate and chirality for DWNTs might become more complicated. Besides, due to the similar function and same wall number, other species within s‐DWNT populations are possible to transform into the near‐(2*n*, *n*)‐containing types with greater kinetic stability, while the faraway Euclidean distances from s‐DWNTs to other CNT populations almost eliminate the possibility of transformation across populations. This may partly account for the increase of s‐DWNT abundance within the 19.1 ± 2° range at 70 mm in the statistics of the chirality distribution. The superiority of growth rate and the potential transformation of chirality are significant topics deserving deeper explorations, so that it can provide more perspectives for understanding the evolutionary growth mechanism of CNTs.

**Figure 4:**
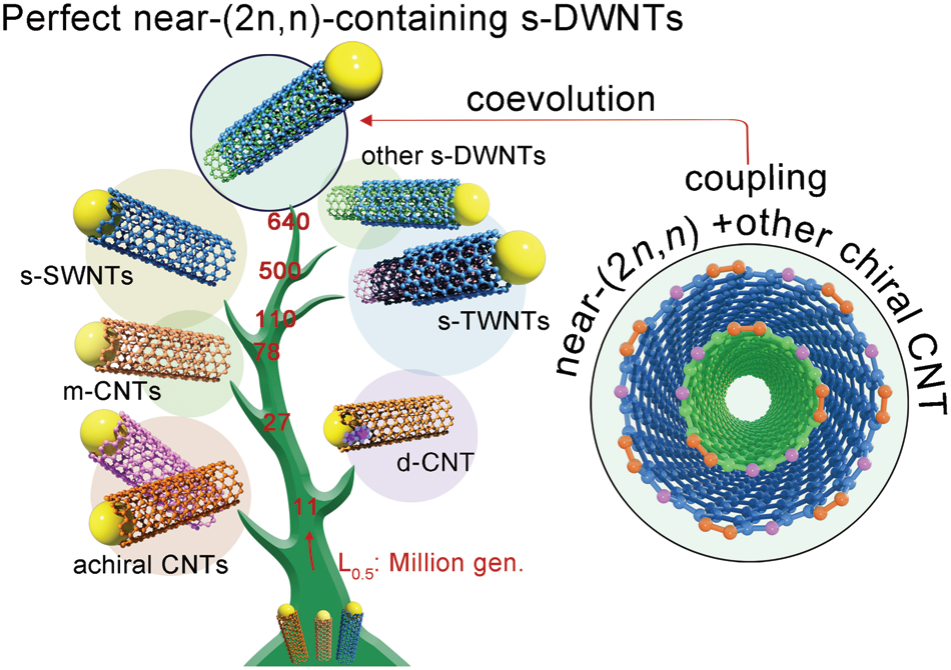
Schematic summarizing the stepwise evolution toward perfect near‐(2*n*, *n*)‐containing semiconducting DWNTs. The evolutionary trend is depicted in the form of evolutionary tree, and the positions of different branches are based on the number of “generations” corresponding to the half‐length *L*_0.5_, noted as “gen.”. Achiral CNTs are primarily driven to extinction, followed by the d‐CNTs, while m‐CNTs are inferior to s‐CNTs. For s‐CNTs with different wall numbers, s‐SWNTs and s‐TWNTs are eliminated successively, less advantageous in survival than s‐DWNTs, among of which the s‐DWNTs containing at least one near‐(2*n*, *n*) layer are the most preferred, supported by inter‐wall coupling and molecular coevolution.

The clear trend toward perfect s‐DWNTs containing near‐(2*n*, *n*) component can be integrally summarized as the evolutionary tree in **Figure** **4**. Firstly, the achiral CNTs, which are featured without helix structure and hardly formed in general, will promptly be eliminated in the evolutionary growth due to their obvious kinetic inferiority, then the d‐CNTs with destroyed atomic and bandgap structure. Due to the bandgap‐coupled assembly kinetics, m‐CNTs are less preferential than s‐CNTs. Further evolutionary competition within the s‐CNTs come from the difference of wall number, where s‐SWNTs and s‐TWNTs are inferior to s‐DWNTs in the evolution. Among s‐DWNTs, the ones containing at least one near‐(2*n*, *n*) layer are the most preferred, supported by inter‐wall coupling and molecular coevolution. Concretely, the (2*n*, *n*) species are the chiral types with the highest configurational entropy.^[^ ^37^ ^]^ Thus, in the coevolution of DWNT, the near‐(2*n*, *n*) layer is just like the “wild type” describing the ordered phase of matter in the molecular evolution.^[^ ^38^ ^]^ Besides, the coevolution demands the necessary existence of another disordered phase that is strongly competitive yet closely coupled with it to constitute a stable system.^[^ ^29^ ^]^ The coupling of near‐(2*n*, *n*) species and other chiral layers would endow the coevolution of DWNTs with greater kinetic stability.

### 2.4 Environmental Effect on the Evolutionary Growth

In fact, aforementioned growth results are based on mixed carbon source involving ≈1 vol% C_2_H_2_ and ≈99% CH_4_. The details about the reason why the mixed carbon source was adopted instead of pure CH_4_ and the comparison between growth results with different carbon sources are presented in Sections S5–S8 and Figures S8–S11, Supporting Information. When using pure CH_4_, evolutionary growth trend was also observed, but within a different kinetically‐stable space. The chiral indices of s‐DWNT walls are almost evenly distributed along the (2*n*, *n*) and (*n*, *n*‐1) lines (Figure S12, Supporting Information). Besides, the mixed carbon source yields CNTs with evidently‐smaller CNT diameter in a narrower distribution, typically 2.13± 0.21 nm (Figure S13, Supporting Information). Given the inverse relation between bandgap and diameter for s‐CNTs, the molecular evolutionary growth of CNTs with mixed carbon source was advantageous for the larger‐bandgap CNT‐based electronic devices (Sections S9 and Figure S14, Supporting Information).

The evolutionary growth can be demonstrated in both situations with the two kinds of carbon sources. Some selective preparations of (2*n*, *n*) species have been achieved previously for micrometer‐length‐level SWNTs grown with solid catalysts, based on the methods of thermodynamics‐dominated match and selection.^[^ ^35^, ^36^, ^39^ ^]^ Compared with these investigations, the kinetic competition in the TAC assembly, as well as the inter‐wall coevolution, is sufficiently displayed in our growth results, which means the evolutionary growth of doubled‐walled s‐CNTs with near‐billion generations of assembling iterations. More significantly, our experimental results with different carbon sources demonstrate that such a tiny difference in the environment could primarily alter the dominant assembling pathways and corresponding chirality distribution, which indicates that the dominant species should be more adaptive to specific environment.

Besides the types of carbon sources, more factors associated with the environment will affect the evolutionary growth. For example, the composition and content of the reaction atmosphere play a critical role in determining the direction of evolutionary growth, regardless of the physical state of carbon precursors. Moreover, temperature is another important environmental factor in an immaterial form. Previous investigations revealed that the temperature would alter the diameter,^[^ ^40^ ^]^ as well as the distribution of diameter and wall number^[^ ^41^ ^]^ of the as‐prepared CNTs. All these investigations demonstrated the effect of external environmental factors on the evolutionary growth. Due to the same mechanism of template auto‐catalysis assembly, the evolutionary growth trend of ultralong CNTs would be valid under different growth conditions, although the evolutionary rate, evolutionary direction, and the resultant survival species would differ.

## 3 Conclusion

Based on the demonstrations from different aspects, the understanding for the evolutionary growth of CNTs gets gradually explicit. The kinetic growth of ultralong CNTs follows the template auto‐catalysis and far‐from‐equilibrium assembly principles. The growth based on atomic assembly is dominated by kinetics and boosted by auto‐catalysis loop with positive feedback to obtain an ultrafast rate, which builds a basis for the far‐from‐equilibrium principle. Meanwhile, the atomic templates ensure that CNTs can be assembled into defined structures autonomously, making the positive feedback conducted in the direction toward entropy reduction of the carbon structure. These principles are not contradictory but interdependent, collectively representing the intrinsic powers that endow the growth with evolutionary features, while the effect of external environment on the evolution is also considerable.

Moreover, the breaking of chiral symmetry usually accompanies with the chirality‐involved TAC,^[^ ^42^, ^43^ ^]^ which means the products become enantioselective as the assembly proceeds, mostly concentrated on left‐handed or right‐handed chirality. It is typically associated with natural homochirality in bio‐systems. For the evolutionary growth of ultralong CNTs, we anticipate the chiral symmetry would break after specific length, which deserves deep investigations.

We demonstrate the molecular evolution based on TAC mechanism in the growth of ultralong CNTs, providing a distinctive viewpoint to understand the growth and promote precisely selective preparation of chiral materials. Furthermore, CNT is a simple and clear platform for investigating molecular evolution. The outstanding kinetic stability of ultralong CNTs could motivate related research fields, to utilize molecular evolution and develop template self‐assembling preparation systems with more enduring survival to perform excellent properties.

## 4 Experimental Section

### Preparations of Ultralong CNTs in Tubular Furnace

The as‐prepared ultralong CNTs were catalyzed by Fe which was predispersed in the ethanol solution (0.03 mol L^−1^) and dipped onto the Si/SiO_2_ substrate being cut into rectangle strips. In some situations, the Si/SiO_2_ substrates with special symbol marks fabricated by photolithography and dry etching were used to help locate as‐grown CNTs.

The CVD process involves reduction, reaction and cooling stages. The temperature was increased stepwise in a reductive atmosphere of H_2_/Ar (*V*_H2_ : *V*_Ar_ = 2:1 with a total flow of 150 sccm) to 1005 °C and kept for 20 min. The carbon source, H_2_ and H_2_O (*V*_H2_ : *V*_carbon‐source_≈2:1–2.5:1, the total flow is 75 sccm with a trace amount of H_2_O vapor) was used as reaction gas flow. The CH_4_ and C_2_H_2_ are premixed with a volume ratio about 1:99 to serve as mixed carbon source. In some situations, pure CH_4_ is used as carbon source. After a reaction segment of 10–50 min, the gas was switched to H_2_/Ar until it cooled to room temperature. The exhaust gas of CVD was analyzed by an in situ quadrupole mass spectrometry (Hiden Analytical QIC20).

### Preparations and Visualization of Wafer‐Scale Ultralong CNTs

The wafer‐scale preparations of ultralong CNTs were conducted in the muffle furnace, and the process is similar to the above descriptions. Sequentially placed silicon wafers were loaded on a quartz plate and encapsulated into the home‐made layered rectangular reactor. The catalysts were deposited with the preloading strategy.^[^ ^44^ ^]^ The CNT growth process resembled the above demonstrations except that the gas composition was *V*_H2_: *V*_carbon source_ ≈2:1–2.5:1 with a total flow of 154 sccm and ≈0.5% H_2_O content. To optically visualize the CNTs on the substrate, a commercial humidifier was reformed with a metal joint and a hosepipe to produce oriented vapor. Illuminated under the light, a bottle of liquid nitrogen was put under the substrates, then the vapor was blown towards the silicon wafer to make the CNTs visible with naked eyes.

### Preparation of Air‐Suspended CNT Arrays and Its Optical Visualization

The substrates with home‐made (depth: 0.25–0.5 mm; width: 1–7 mm) trenches could be used to prepare air‐suspended CNT arrays according to the aforementioned CVD process. Then a process to deposit TiO_2_ particles onto the air‐suspended CNT arrays were conducted to make the arrays optically visualized. Further details can be found in a previous work.^[^ ^45^ ^]^

### Characterizations of Ultralong CNTs

Scanning electronic microscope (SEM, JSM 7401F, 1.0 kV), transmission electronic microscope (TEM, JEM 2010, 120 kV), and atomic force microscope (AFM, Asylum Cypher) were used to inspect the morphology and structure of ultralong CNTs. Raman spectrometer (Horiba HR 800, 532/633/785 nm) was used to characterize the structural defects and metallicity, by distinguishing D band and G band. An optical microscope (long working distance metallography microscope, FS 70Z) and supercontinuum laser (Fianium SC‐400‐4) were utilized for resonant Rayleigh scattering (RRS). The Raman and Rayleigh optical characterizations were conducted after the samples were annealed in Ar atmosphere (450 °C) for 15 min, so that the optical signal of intrinsic CNTs could be detected with all the adsorbed amorphous carbonaceous impurities and O_2_ molecules removed. Besides, the sample was encapsulated in a stage (THMS600E, Linkam Scientific Instrum., the U.K.) for Raman measurements. More experimental details about Raman characterizations can be seen in the previous investigations.^[^ ^18^ ^]^ CNTs on Si/SiO_2_ substrate were transferred onto copper grids using cellulose acetate film, for TEM characterization.^[^ ^41^ ^]^

### Fabrication and Measurement of the Transistors

Ultralong CNTs grown on Si/300 nm SiO_2_ substrate was first annealed in Ar atmosphere (450 °C) to remove the adsorbed amorphous carbonaceous impurities. Pd film of 70 nm was then patterned on the substrate using electron beam lithography and deposited via electron beam evaporation under high vacuum, followed by the deposition of Ti/Au (5/40 nm) source/drain metal electrodes with a similar process as Pd. Electronic measurements were carried out by applying drain and gate voltages relative to the source electrode with a Keithley 4200 A parameter analyzer at room temperature in air.

## Conflict of Interest

The authors declare no conflict of interest.

## Supporting information

Supporting Information

Supporting Information

## Data Availability

The data that support the findings of this study are available in the supplementary material of this article.
