# Supplementary material for: Molecular Evolutionary Growth of Ultralong Semiconducting Double‐Walled Carbon Nanotubes
Source: Adv Sci (Weinh). 2022 Nov 24;10(1):2205025. doi: 10.1002/advs.202205025 (PMC9811487; doi:10.1002/advs.202205025)
Supplement: Supplementary file 1 — Supporting Information [file ADVS-10-2205025-s002.pdf]

ADVANCED SCIENCE
Open Access

Supporting Information

for *Adv. Sci.*, DOI 10.1002/advs.202205025

Molecular Evolutionary Growth of Ultralong Semiconducting Double-Walled Carbon Nanotubes

Jun Gao, Yaxin Jiang, Sibo Chen, Hongjie Yue, He Ren, Zhenxing Zhu and Fei Wei**

Supporting Information for

Molecular Evolutionary Growth of Ultralong Semiconducting Double-Walled Carbon Nanotubes

Jun Gao, Yaxin Jiang, Sibo Chen, Hongjie Yue, He Ren, Zhenxing Zhu*, Fei Wei*
Correspondence to: Zhenxing Zhu, zxing@mail.tsinghua.edu.cn; Fei Wei, wf-dce@tsinghua.edu.cn

This PDF file includes:

Supporting Sections 1 to 9
Supporting Figures 1 to 14
Supporting Tables 1 to 6
Captions for Supporting Data 1

Other Supporting Materials for this manuscript include the following:

Supporting Data 1

Table of contents

Supporting Section 1 4

Supporting Section 2 4

Supporting Section 3 5

Supporting Section 4 6

Supporting Section 5 6

Supporting Section 6 6

Supporting Section 7 7

Supporting Section 8 8

Supporting Section 9 9

Figure S1 10

Figure S2. 11

Figure S3. 12

Figure S4. 13

Figure S5. 14

Figure S6. 15

Figure S7.. 16

Figure S8. 17

Figure S9. 18

Figure S10. 19

Figure S11.. 20

Figure S12 21

Figure S13. 22

Figure S14. 23

Supporting Table 1 24

Supporting Table 2 25

Supporting Table 3 26

Supporting Table 4 27

Supporting Table 5 28

Supporting Table 6 31

Supporting Data 1 (separate file) 34

References 35

Supporting Section 1. Perfect structure of as-prepared ultralong CNTs

In Rayleigh resonance scattering (RRS), a single carbon nanotube (CNT) owning a change in chirality or structure along its axial direction would show a color change accordingly,[1] which provides an efficient method to identify the chiral consistency.[2, 3] The monochromatic characteristics at different positions of the CNTs on the substrate, as well as the consistent energy values of RRS peaks, elucidate the chirality remains consistent along the axial direction. The air-suspended CNTs that exclude the influence of substrate also behave homogeneous color in the RRS images, demonstrating the reliability of monochromatic characteristics (Figure S2). Furthermore, the high density of air-suspended CNT arrays and the ability of crossing even 6-mm-wide trenches in the flying-kite growth, can also prove the growth stability and perfect structure of the CNTs (Figure S4).

Supporting Section 2. The calculation of the number of 'generations' based on the length of ultralong CNTs

Each round of circumferential atomic assembly during the CNT growth can be regarded as the production of a new 'generation'. For CNTs, each round of circumferential atomic assembly yields a length increment in the direction of the tube axis, which can be described as (Figure S5):

$$L_{each-round} = \sqrt{3}a_{C-C} * \cos(30^{\circ} - \theta)$$

where θ is the chiral angle, a_{C-C} is the distance between neighboring two carbon atoms in CNTs and is usually considered as 0.14 nm.

The value of $\cos(30^\circ - \theta)$ falls in the range from $\frac{\sqrt{3}}{2}$ to 1 in the whole range of chiral angle θ. As the range from $\frac{\sqrt{3}}{2}$ to 1 is not large and the length of nascent seeds for ultralong CNTs can be neglected, thus the number of 'generations' for ultralong CNTs can be calculated as $\frac{L}{0.5*(\frac{\sqrt{3}}{2}+1)*\sqrt{3}a_{C-C}}$, where L is the length of ultralong CNT.

Supporting Section 3. The calculation of Euclidean distances for different types of CNT

We adopt Euclidean distance, a valid and widely-used parameter for characterizing the divergence among different species or populations in biology and ecology,[4, 5] to measure the evolutionary divergence among different kinds of CNTs. Different types of CNTs contain the ones decaying in different length interval, which represents the survival time and iterative generations. Thus, the Euclidean distances in this work were calculated by:

$$D_{12} = \sqrt{\sum_{j=1}^{p}(N_{1j} - N_{2j})^2}$$

where D_{12} is the Euclidean distance between the CNTs of type 1 and type 2, N_{1j}, N_{2j} are the number density of CNTs decaying in the length interval *j*, and *j*=1 to *p* are the length intervals where we collected the statistics of number density *N*.

For example, the Euclidean distance between s-CNT and m-CNTs was calculated based on the number density listed in Supporting Table 1, as:

$$D = \sqrt{(2.34-1.34)^2 + (0.72-0.15)^2 + ... + (0.40-0.00)^2 + (2.20-0.00)^2} = 2.65$$

And the result is shown in Supporting Table 2. Similarly, the number density and the as-derived Euclidean distances are shown in Supporting Tables 1 to 4.

Supporting Section 4. Identify the chiral indices by combing RRS and Raman spectra

Combining RRS with Raman spectra could help to identify chiral indices of CNTs, with higher reliability and accuracy. Based on the resonance peaks resolved from RRS spectra, we can identify the energy resonant with CNTs and choose an appropriate laser to conduct Raman spectroscopy characterizations. The resonant energy values and RBM positions of Raman spectra under different excitation wavelengths can be used to assign the chiral indices of CNTs by consulting the atlas,[6] while the shape of G band would also help distinguish s- and m-CNTs.

Supporting Section 5. Different segments of operating process of chemical vapor deposition (CVD) preparation

The actual operating process of CVD preparation can be mainly divided into reduction segment, reaction (growth) segment and cooling-down segment. The setups of temperature and gas flow in each segment are schematically shown in Figure S8, to help understand the *in-situ* mass spectra in different stages.

Supporting Section 6. The analyzation for in-situ mass spectra to identify the key role of C_2H_2

We used *in-situ* mass spectrometry to monitor the atmosphere and identify key factors in the CVD process.

Typical *in-situ* mass spectra in the growth with pure CH_4 are shown in Figure S9c. In the reduction segment, the air in the reactor has been extruded out, verified by the blank at mass number m/z = 28, 29 that indicates N_2 or C_2H_4. Then in the reaction segment, the signal at m/z

= 28 could confirm the existence of C_2H_4. And the relative weaker signal at *m/z* = 29 due to the isotopic distribution can also be identified as C_2H_4. Furthermore, a small amount of C_2H_2 was found at *m/z* = 26 and 27. The relative intensity of C_2H_4 and C_2H_2 peak is also consistent with previous research.[7]

Furthermore, the growth states with different CVD parameters are related to the intensity of C_2H_2 and C_2H_4 peak. Specifically, CNTs decayed less rapidly in batch 1, with a number density *N* at 15-mm-length position of 2.54/100 μm, much higher than the 1.28 for batch 2 (Figure S9, a and b). While the C_2H_2 and C_2H_4 peak intensity (Figure S9c) and the intensity variation from the reduction segment to the reaction segment (Figure S9d) in batch 1 were also higher. Then we normalized the total peak area of C_2H_2 and C_2H_4 based on the peak area of Ar. Finally, we quantified the number density at 15-mm-length position versus the normalized total peak area of C_2H_2 and C_2H_4, respectively (Figure S9e). The number density increases with the peak area of C_2H_2 and C_2H_4, behaving positively-related dependences. While exerting similar effect on the growth, the total amount and the variation of C_2H_2 are also much smaller than C_2H_4. These phenomena and the high-energy feature of C_2H_2 demonstrate that C_2H_2 is of greater significance to the out-of-equilibrium template auto-catalysis (TAC) growth. Besides, alkynes have been shown to accelerate the growth of vertically-aligned CNTs and thought to be related to the auto-catalysis.[8] Thus, introducing a small amount of C_2H_2 is necessary to investigate its effect, without drastic alteration to the original carbon source.

Supporting Section 7. The enhancement effect of the introduction of a minor amount of C_2H_2

The mass spectra revealed that the amount of C_2H_2 is about 1% of CH_4, while exerting obvious effect on the growth. Therefore, we used ~1% C_2H_2 +~99% CH_4 as the mixed carbon

source. The experimental results demonstrated it could prominently enhance CNT growth, further confirming the key role of C_2H_2.

Firstly, the effective introduction of C_2H_2 and its volume percentage are verified by the mass spectra in cold state (Figure S10a) and actual growth (Figure S10b). The elongation growth with the mixed carbon source also owns a constant rate, slightly larger than that of CH_4 (Figure S10c).

A typical SEM image of ultralong CNT arrays prepared with mixed carbon source (Figure S1a) manifests the parallel morphology, possessing a number density of 4.32/100 μm at 15-mm-length position, exceeding that in Figure S9a by a factor of ~1.7. Besides, representative large-range overviews for as-prepared CNTs demonstrated the number density and growth persistence were enhanced by the introduction of a trace of C_2H_2 (Figure S11).

The modified CVD growth involves ~1% vol substitution of C_2H_2, but much larger volume ratios of H_2/mixed carbon source are found to be more advantageous for growth, thus the atom ratios of H/C are also increased. Therefore, the amount of available carbon atoms could not account for the unexpected increase of number density. It demonstrated the key role of C_2H_2, the direct supply of which with tiny amount can obviously promote the out-of-equilibrium auto-catalysis of CNTs, compared with the pure CH_4 situation, where C_2H_2 must be provided indirectly after the conversion.

Supporting Section 8. The evolutionary growth with pure CH_4 carbon source

In the growth with pure CH_4 as carbon source, the chirality distribution also behaves an evolutionary trend. The chiral indices of CNTs are randomly distributed at first. As length increases, an obvious phenomenon of gathering along two discrete lines of ($2n$, n) and (n, n-1) appears, and the chiral indices are concentrated on fewer species gradually. The chirality

distributions at multiple length positions are summarized in Figure S12. For example, at the position of 80 mm length, the abundance of chiral angle within 19.1° ± 5° reaches 40.4%. Moreover, (n, n-1) and (n, n-2) species achieve a combined abundance of 56.3%. The distribution is almost balanced for the two lines. The gradually-decreased entropy can also be attributed to the continuous energy input, under the TAC mechanism.

Supporting Section 9. Representative performances of the as-manufactured CNT transistors

The excellent structural and electronic properties of as-prepared ultralong CNT arrays with mixed carbon source, as well as the relatively smaller diameters, bring about exceptional performance of the manufactured transistors. The transfer characteristics curves (I_{DS} -V_{GS}) measured at V_D=-2 V bias of four devices (Figure S14) all exhibit excellent electrical performances, such as the on/off ratio of around 10^5, even 10^7, on-state current about 5 μA.

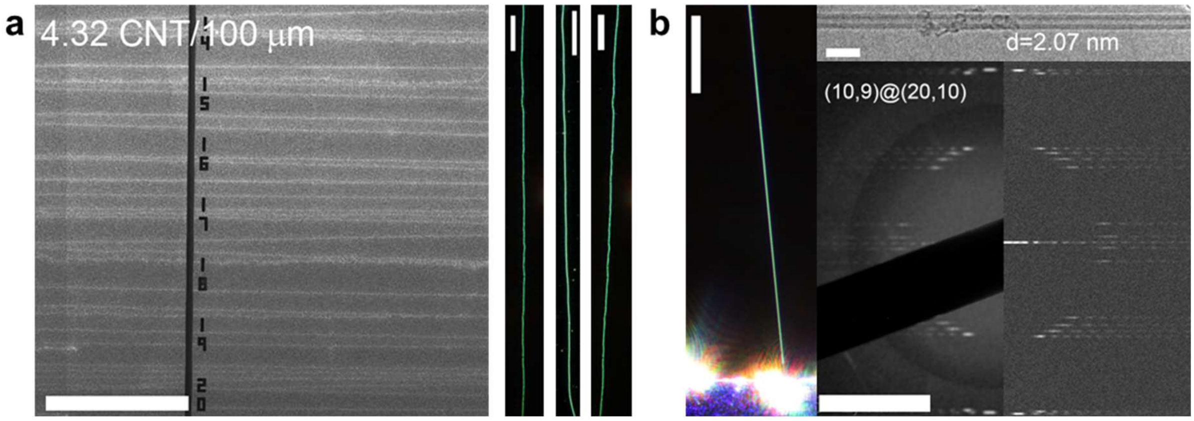

Figure S1. Parallel array morphology and perfect structure of as-prepared CNTs. a) A typical SEM image of the as-prepared ultralong CNT arrays at the position of 15-mm length, showing a CNT number density of 4.32/100 μm. Three true-color RRS images along the length direction show consistent color. b) A typical true-color RRS image of an air-suspended ultralong CNT prepared with mixed carbon source, showing consistent color along the length. The glaring light at the bottom is the edge of the substrate under the illumination of laser. Its TEM characterization and corresponding electron diffraction pattern identify it to be a (10, 9)@(20, 10) double-walled CNT, by comparing it with the simulation result. Scale Bars: SEM 500 μm, RRS images 100 μm (a); RRS image 100 μm, TEM 5 nm, electron diffraction image, 1/5 1/nm (b).

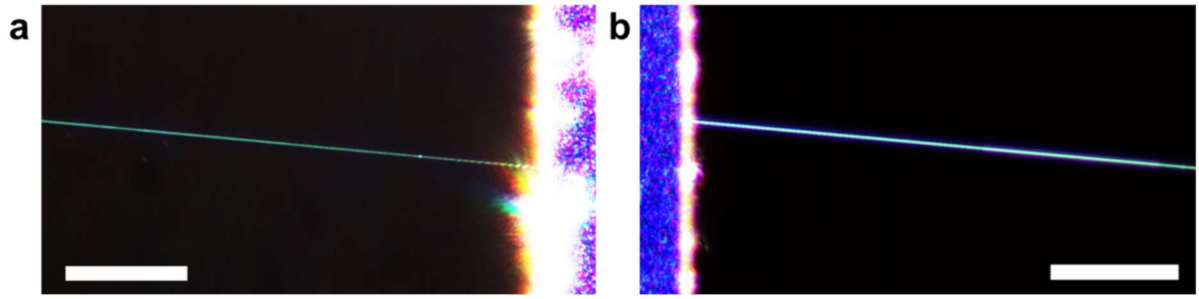

Figure S2. Typical true-color resonance Rayleigh scattering images of two air-suspended ultralong CNTs. The images all show consistent color along the length. The brighter spot in the middle of the CNT is an adhering particle from air, the glaring lights are the edge of the substrates under the illumination of supercontinuum laser. Scale bars: 100 μm.

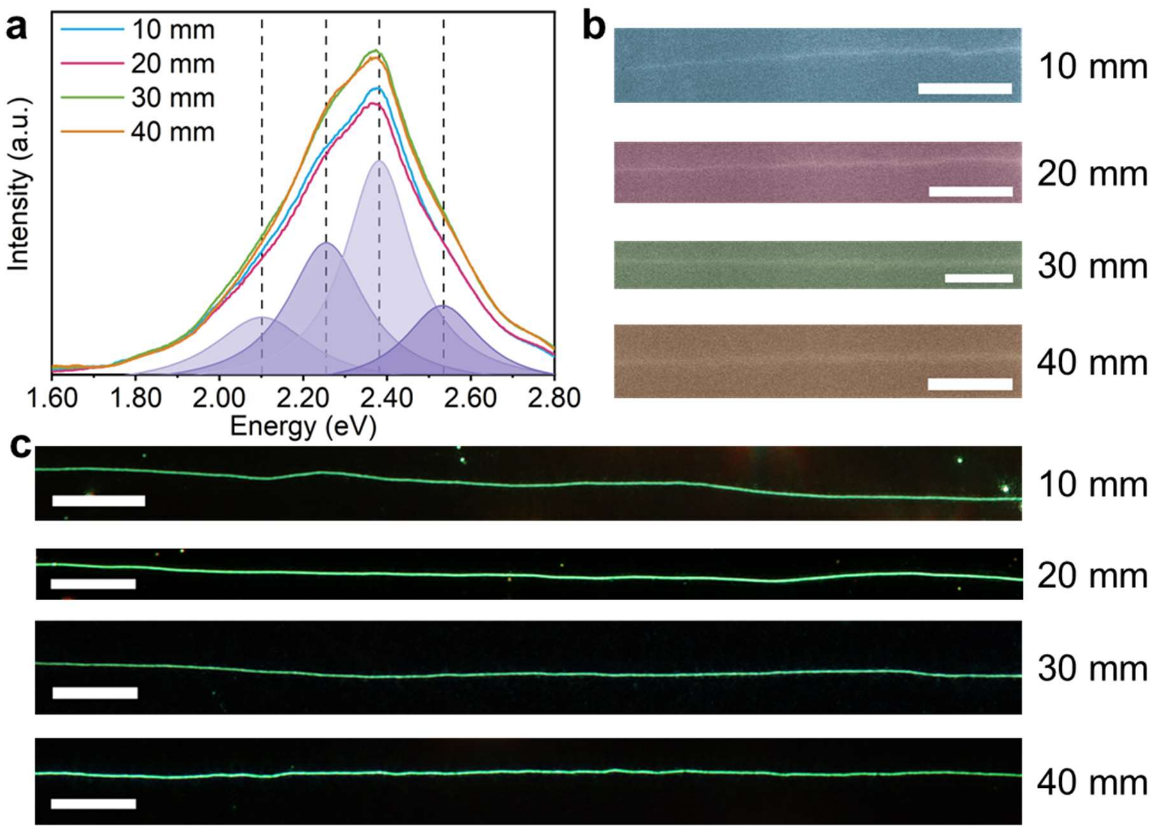

Figure S3. Consistent structure of CNTs along the length verified by RRS images and spectra. a) RRS spectra, b) SEM images and c) true-color RRS images obtained at the different positions along an ultralong CNT prepared by the mixed carbon source. The peak positions of Rayleigh scattering spectra and the color of the CNT are consistent. Scale bars: 200 μm (b), 100 μm (c).

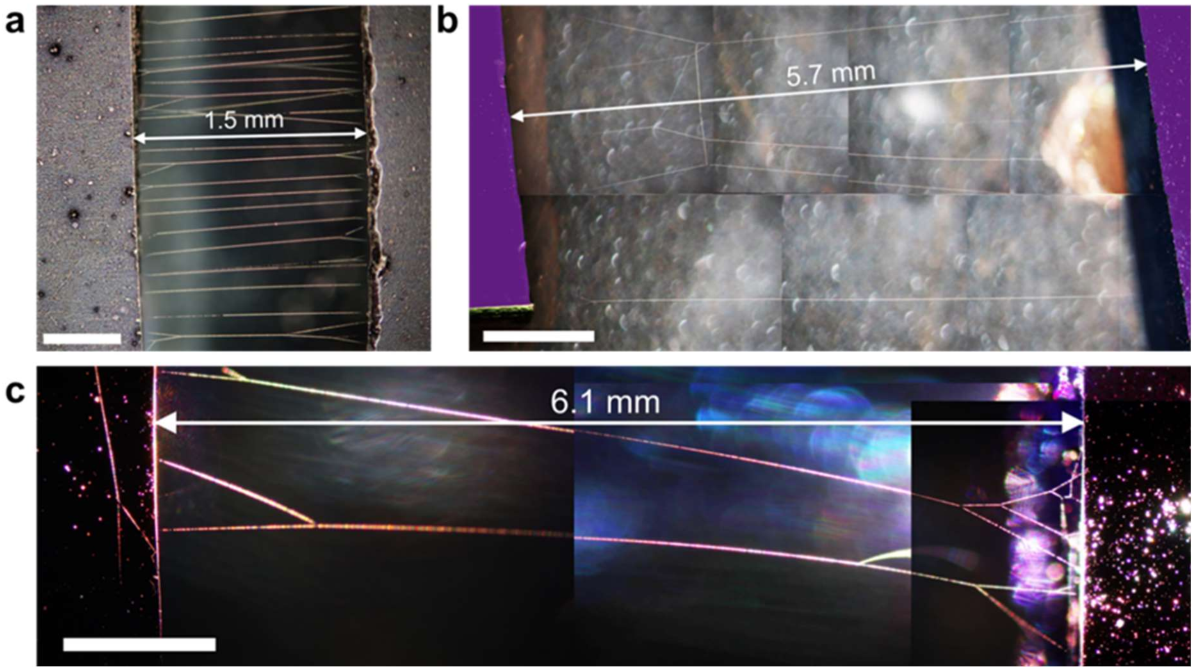

Figure S4. The growth stability verified by TiO_2-assisted optically visualized air-suspended CNT arrays. a) The high density of air-suspended CNT arrays. b, c) The ability of crossing even 6-mm-wide trenches in the flying-kite growth, which can also indicate the perfect structure of as-prepared CNTs. Scale bars: 500 μm (a); 1 mm [(b) and (c)].

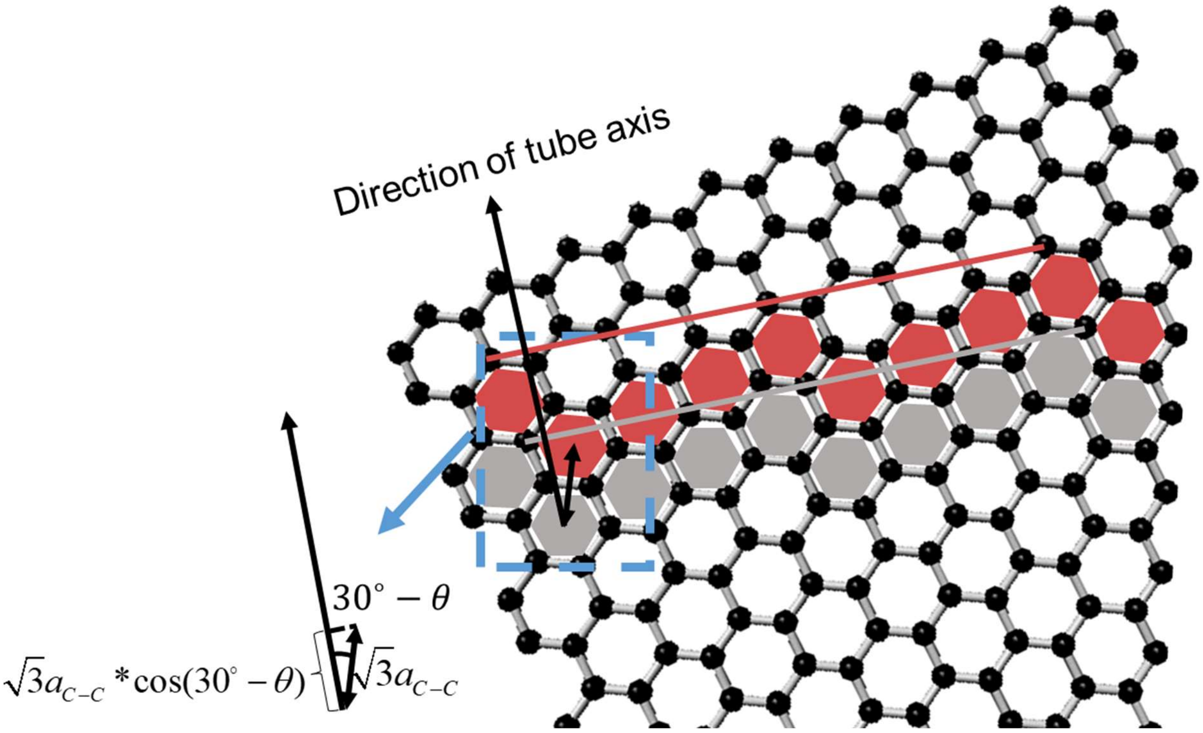

Figure S5. Schematic of a CNT to illustrate the length increment yielded by each round of circumferential atomic assembly. It can further assist in calculating the number of iterative generations. The hexagons in gray and carmine represent the CNT edges in the last round and the new round, respectively.

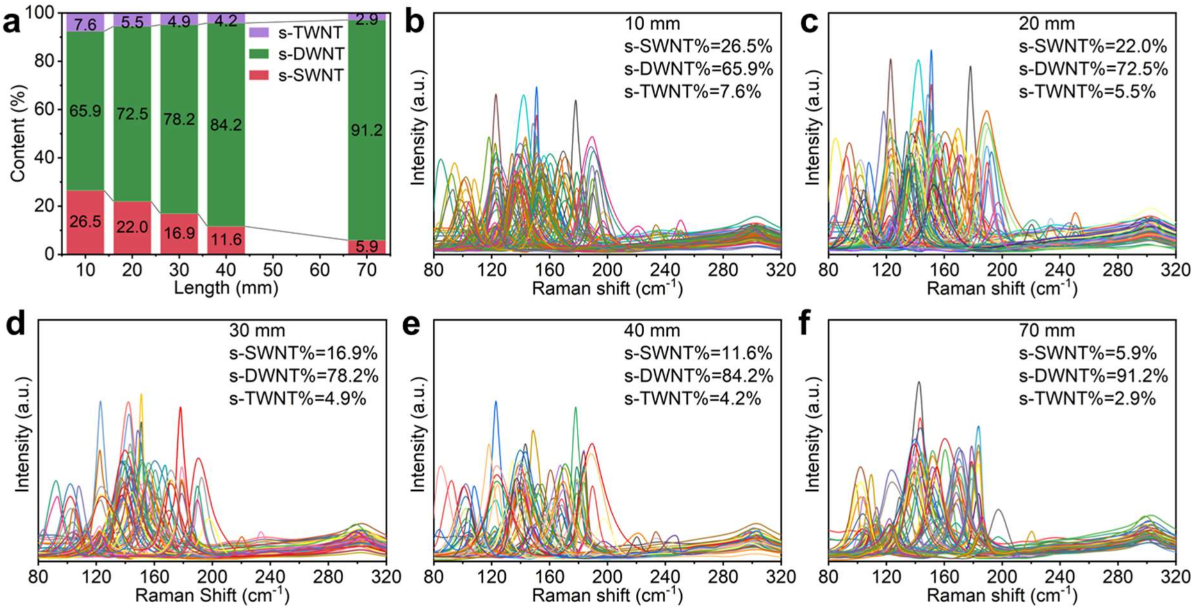

Figure S6. The evolution of CNTs with different wall numbers. a) The variation of the content of CNTs with different wall numbers as length increases, under the CVD situation with mixed carbon source. b-f) The RBM peaks of Raman spectra at multiple positions with the length of 10 mm (b), 20 mm (c), 30 mm (d), 40 mm (e), 70 mm (f).

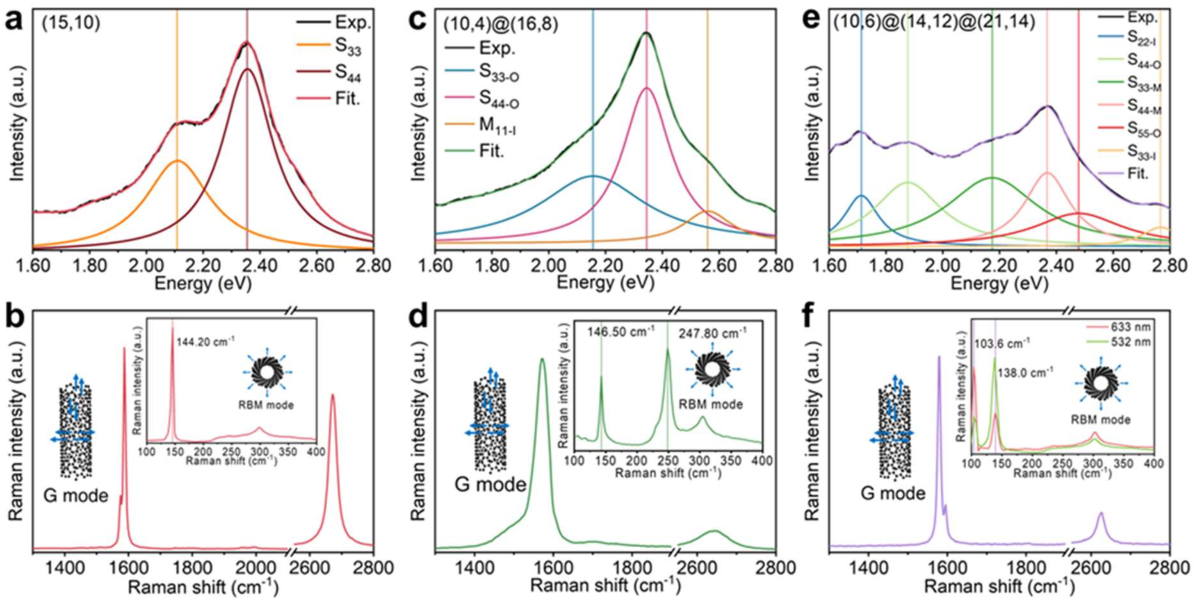

Figure S7. Typical RRS spectra and corresponding Raman spectra to identify the chiral indices. The RRS and Raman spectra for SWNT (a and b), DWNT (c and d), TWNT (e and f) are exhibited, respectively. The insets in (b), (d), (f) are RBM mode range of Raman spectra.

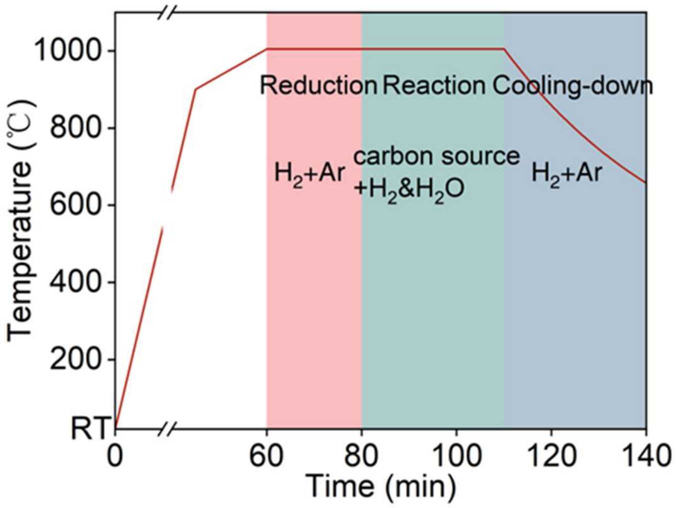

Figure S8. Schematic for the CVD operating process. It shows the temperature and gas inlet for reduction, reaction and cooling-down segments, to help understand the *in-situ* mass spectra in different stages.

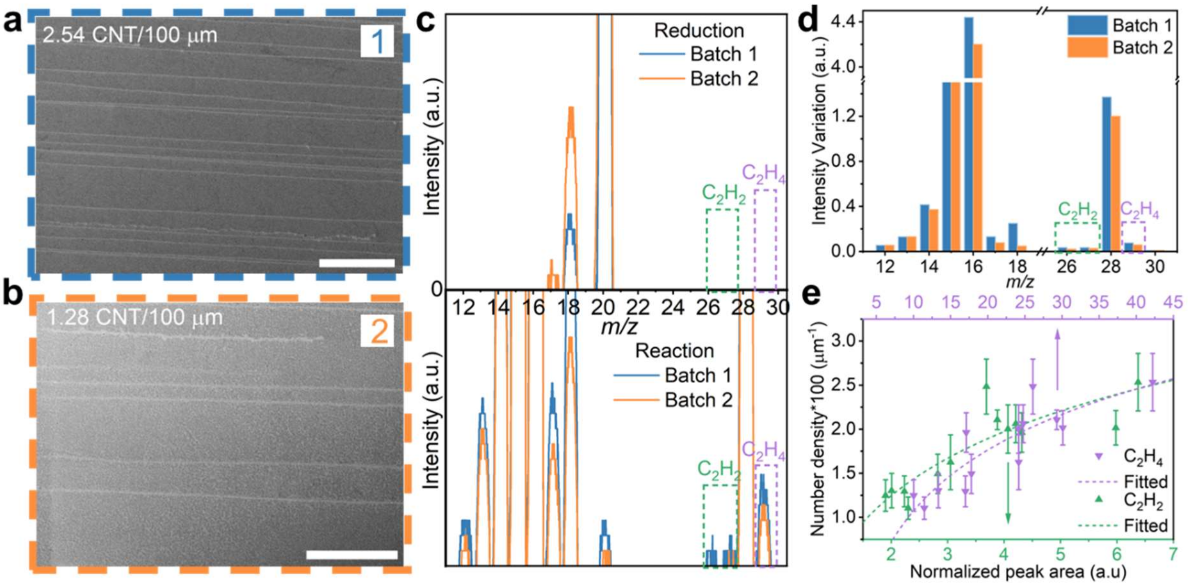

Figure S9. Identification of the important role of C_2H_2 in the CNT growth with pure CH_4 carbon source. a-c) Comparison of the SEM images (a and b) and mass spectra (c) for two representative growth batch, noted as batch 1 and batch 2. A SEM image of batch 1 (a) shows the CNT number density of 2.54/100 μm at the position of 15 mm length, compared with that of 2.54/100 μm for batch 2 (b). c) Mass spectra of reduction segment (upper panel) and reaction segment (lower panel) of batch 1 and 2, showing different features. d) The intensity variation of the peaks from reduction segment to reaction segment for batch 1 and 2, showing the amount increases of C_2H_4 and C_2H_2 are larger in batch 1 than batch 2. e) The relationship between the CNT number density at the position of 10 mm length and the normalized peak area summation of C_2H_4 and C_2H_2 in the whole reaction segment, respectively. The data come from different growth batches and the error bars come from the number density statistics. Scale bars: 500 μm [(a) and (b)].

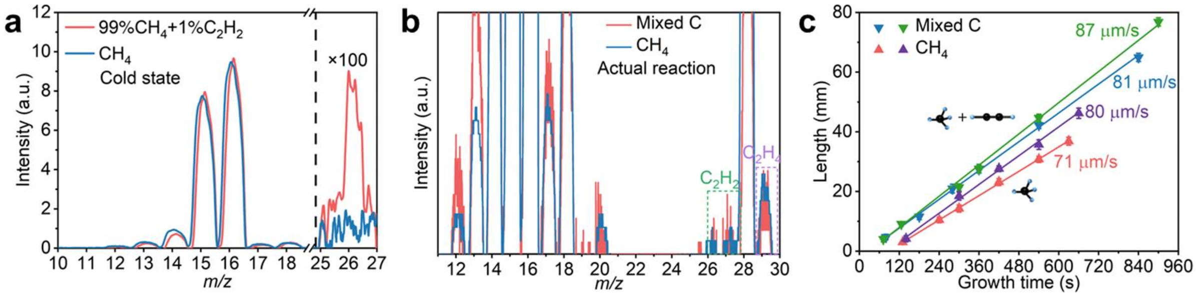

Figure S10. The comparison of mass spectra and growth rate between different carbon sources. a) Comparison of mass spectra for the situations of pure CH_4 and mixed carbon source, at the cold state, i.e., only conducting the procedure of gas flow inlet without heating. b) Comparison of mass spectra in the reaction segment of actual growth, for the situations of pure CH_4 and mixed carbon source. c) The average CNT length plotted against the growth time, for the situations with traditional pure CH_4 and mixed carbon source. For CNTs with different length on substrates, only the longest 10 CNTs were taken into consideration for calculating the growth rate. Error bars represent standard deviation of the length for CNTs.

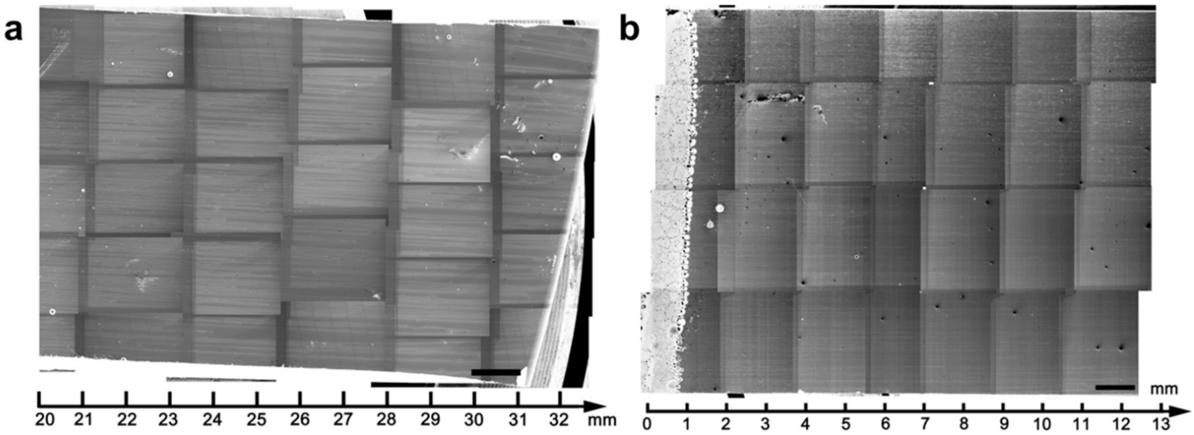

Figure S11. The growth enhancement effect of mixed carbon source verified by the overviews of CNT arrays. a) Overview of a typical CNT array prepared with mixed carbon source, in the length range from 20 to 33 mm by combining 30 SEM images, showing a markedly-enhanced array density. b) Overview of another typical CNT array prepared with mixed carbon source, in the length range from 0 to 13 mm. Scale bars: 1 mm.

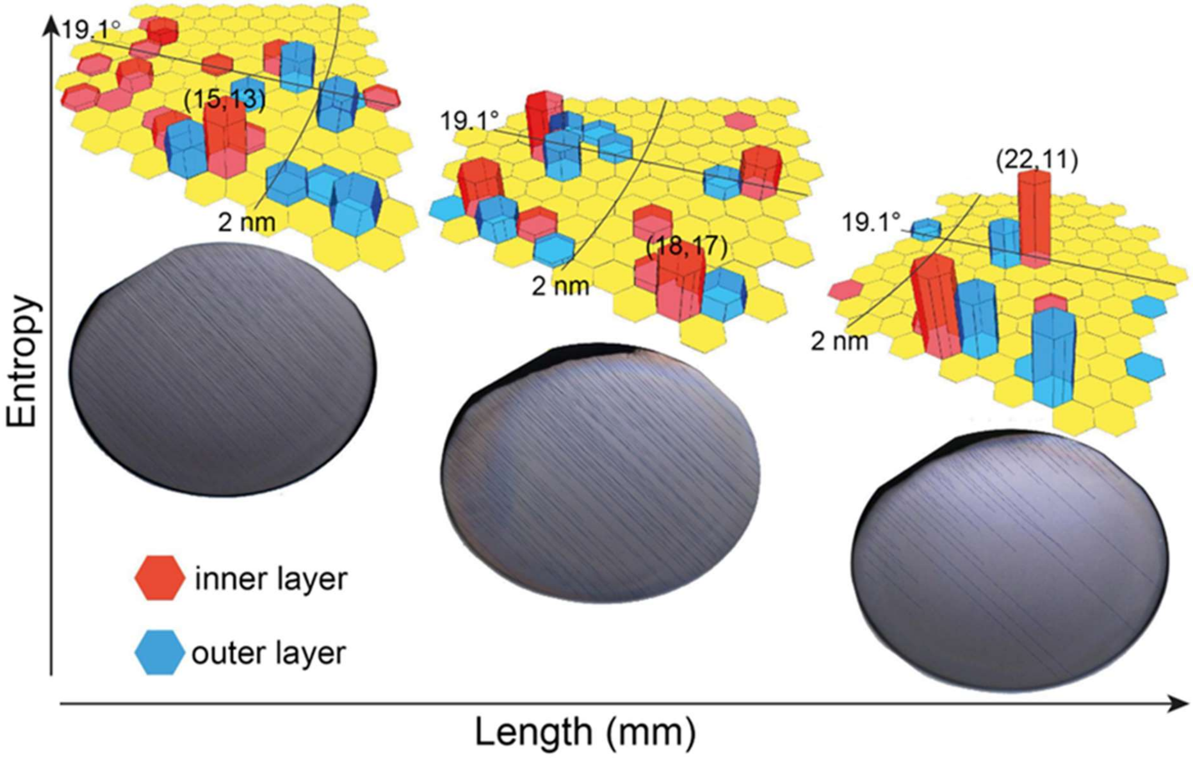

Figure S12. The gradual enrichment in the growth with pure CH_4 carbon source. Chirality distribution of ultralong CNTs at different positions on the 4-inch wafers sequentially placed, with pure CH_4 provided as carbon source. The lines '2 nm', '19.1°' and representative chiral indices are marked to signify the chiral coordinate. The entropy qualitatively represents the diversity of chirality distribution.

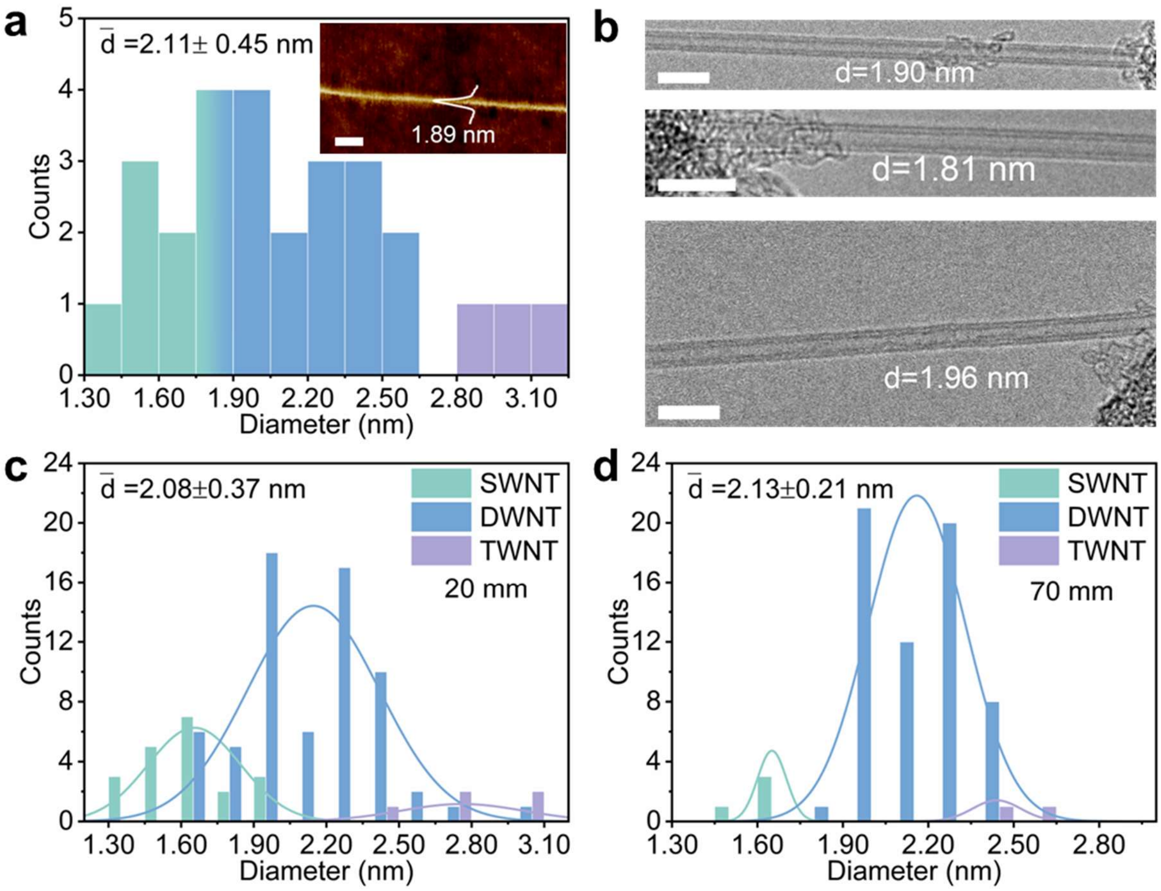

Figure S13. The outer layer diameter distributions of the CNTs prepared with mixed carbon source. a) AFM characterization results of 27 CNTs at 60 mm length for the outer diameter distribution, inset: a typical AFM image of a CNT. b) Three typical TEM images of DWNTs. c, d) Outer layer diameter distributions derived from the chiral indices at 20 mm (c) and 70 mm (d) length positions obtained by Raman and Rayleigh spectra. Scale bars: 20 nm (a); 5 nm (b).

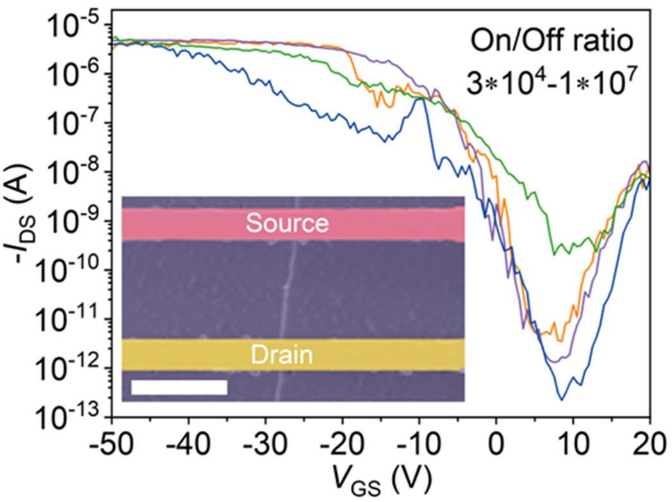

Figure S14. Transfer characteristics of four transistors fabricated based on the CNTs prepared with mixed carbon source. Inset is a typical SEM image of a CNT-based transistor. Scale bar for inset, 5 μm.

Supporting Table 1

Number density (/100 μm) of s-CNTs, m-CNTs and d-CNTs decaying in different length intervals, used to calculate Euclidean distances.

Length interval (mm)	s-CNT	m-CNT	d-CNT
1-10	2.34	1.34	0.16
10-20	0.72	0.15	0.02
20-30	0.35	0.12	0.01
30-40	0.27	0.10	0.00
40-60	0.76	0.04	0.00
60-80	0.48	0.00	0.00
≥80	2.20	0.00	0.00

Supporting Table 2

Euclidean distances of s-CNTs, m-CNTs and d-CNTs (shown in Figure 1h inset).

	s-CNT	m-CNT	d-CNT
s-CNT	0	2.65	3.33
m-CNT	2.65	0	1.20
d-CNT	3.34	1.20	0

Supporting Table 3

Number density (/100 μm) of s-SWNTs, s-DWNTs and s-TWNTs decaying in different length intervals, used to calculate Euclidean distances.

Length interval (mm)	s-SWNT	s-DWNT	s-TWNT
10-20	0.37	0.26	0.14
20-30	0.27	0.12	0.05
30-40	0.23	0.13	0.04
40-70	0.25	0.44	0.07
≥70	0.15	2.26	0.07

Supporting Table 4

Euclidean distances of s-SWNTs, s-DWNTs and s-TWNTs (shown in Figure 2c).

	s-SWNT	s-DWNT	s-TWNT
s-SWNT	0	2.13	0.46
s-DWNT	2.13	0	2.22
s-TWNT	0.46	2.22	0

Supporting Table 5

Chiral indices identified for each wall from the CNTs at the length position of 20 mm, detailed results are shown in Supporting Data 1.

Number	(n, m) for SWNT	(n, m) for outer layer of DWNT	(n, m) for inner layer of DWNT	(n, m) for outer layer of TWNT	(n, m) for middle layer of TWNT	(n, m) for inner layer of TWNT
1	(12,8)	(18,14)	(15,7)	(21,14)	(14,12)	(10,6)
2	(17,6)	(21,14)	(14,12)	(28,11)	(17,10)	(14,4)
3	(15,10)	(21,14)	(16,9)	(26,18)	(22,12)	(14,12)
4	(20,9)	(16,8)	(10,4)	(25,14)	(20,9)	(14,4)
5	(14,7)	(17,7)	(10,5)	(27,17)	(20,14)	(13,11)
6	(17,6)	(20,13)	(17,6)			
7	(16,8)	(22,12)	(18,4)			
8	(22,6)	(20,13)	(17,6)			
9	(12,10)	(21,14)	(16,9)			
10	(11,10)	(18,10)	(13,6)			
11	(17,9)	(22,6)	(12,8)			
12	(17,9)	(16,8)	(9,5)			
13	(14,9)	(21,11)	(14,9)			
14	(17,6)	(15,14)	(14,4)			
15	(15,10)	(18,11)	(12,6)			
16	(14,9)	(18,16)	(16,9)			
17	(12,10)	(19,14)	(14,9)			
18	(20,9)	(22,6)	(13,6)			
19	(15,10)	(22,14)	(14,12)			
20	(12,10)	(16,8)	(9,5)			
21		(18,14)	(15,7)			
22		(21,8)	(14,4)			
23		(21,8)	(11,9)			
24		(22,12)	(17,7)			

Number	(n, m) for SWNT	(n, m) for outer layer of DWNT	(n, m) for inner layer of DWNT	(n, m) for outer layer of TWNT	(n, m) for middle layer of TWNT	(n, m) for inner layer of TWNT
25		(22,12)	(14,12)			
26		(20,9)	(12,7)			
27		(24,11)	(17,9)			
28		(21,14)	(14,12)			
29		(21,9)	(14,7)			
30		(23,13)	(14,12)			
31		(21,8)	(11,10)			
32		(16,8)	(9,5)			
33		(17,10)	(15,2)			
34		(14,12)	(9,7)			
35		(18,16)	(18,4)			
36		(24,20)	(18,16)			
37		(20,16)	(14,12)			
38		(22,12)	(15,10)			
39		(20,9)	(12,8)			
40		(22,12)	(15,10)			
41		(20,16)	(18,7)			
42		(22,6)	(12,8)			
43		(21,10)	(11,10)			
44		(22,11)	(17,6)			
45		(25,12)	(14,13)			
46		(21,10)	(11,10)			
47		(19,5)	(10,5)			
48		(21,11)	(15,7)			
49		(22,17)	(16,13)			
50		(17,9)	(14,4)			
51		(22,6)	(13,6)			
52		(23,4)	(14,4)			
53		(22,11)	(17,6)			

Number	(n, m) for SWNT	(n, m) for outer layer of DWNT	(n, m) for inner layer of DWNT	(n, m) for outer layer of TWNT	(n, m) for middle layer of TWNT	(n, m) for inner layer of TWNT
54		(14,13)	(11,7)			
55		(22,11)	(17,6)			
56		(14,12)	(10,5)			
57		(24,11)	(14,12)			
58		(16,12)	(11,7)			
59		(27,5)	(17,6)			
60		(22,11)	(14,9)			
61		(21,8)	(11,10)			
62		(21,10)	(11,10)			
63		(21,8)	(11,10)			
64		(15,14)	(11,7)			
65		(15,14)	(11,7)			
66		(25,12)	(14,13)			

Supporting Table 6

Chiral indices identified for each wall from the CNTs at the length position of 70 mm, detailed results are shown in Supporting Data 1.

Number	(n, m) for SWNT	(n, m) for outer layer of DWNT	(n, m) for inner layer of DWNT	(n, m) for outer layer of TWNT	(n, m) for middle layer of TWNT	(n, m) for inner layer of TWNT
1	(16,8)	(18,13)	(14,7)	(21,14)	(14,12)	(10,6)
2	(16,8)	(21,14)	(14,12)	(19,18)	(18,8)	(10,6)
3	(15,8)	(21,14)	(16,9)			
4	(15,10)	(22,11)	(17,6)			
5		(22,11)	(17,6)			
6		(21,14)	(16,9)			
7		(18,10)	(13,6)			
8		(22,11)	(14,9)			
9		(18,16)	(16,8)			
10		(18,14)	(14,7)			
11		(22,12)	(14,12)			
12		(18,14)	(15,7)			
13		(21,10)	(11,10)			
14		(22,11)	(17,6)			
15		(22,12)	(14,12)			
16		(24,11)	(17,9)			
17		(21,14)	(14,12)			
18		(21,10)	(14,7)			
19		(22,12)	(14,12)			
20		(21,8)	(11,10)			
21		(20,16)	(14,12)			
22		(21,14)	(15,10)			
23		(20,9)	(12,8)			
24		(22,12)	(15,10)			

Number	(n, m) for SWNT	(n, m) for outer layer of DWNT	(n, m) for inner layer of DWNT	(n, m) for outer layer of TWNT	(n, m) for middle layer of TWNT	(n, m) for inner layer of TWNT
25		(22,12)	(15,10)			
26		(22,12)	(17,6)			
27		(22,11)	(17,6)			
28		(21,11)	(15,7)			
29		(21,11)	(12,11)			
30		(20,16)	(17,9)			
31		(14,13)	(11,7)			
32		(22,11)	(17,6)			
33		(22,12)	(14,12)			
34		(16,12)	(11,7)			
35		(22,12)	(17,6)			
36		(21,12)	(14,9)			
37		(21,10)	(11,10)			
38		(21,10)	(11,10)			
39		(21,10)	(11,10)			
40		(21,10)	(11,10)			
41		(21,10)	(11,10)			
42		(21,11)	(12,11)			
43		(15,14)	(12,7)			
44		(15,14)	(12,7)			
45		(21,11)	(12,11)			
46		(15,14)	(12,7)			
47		(15,14)	(12,7)			
48		(15,14)	(12,7)			
49		(15,14)	(12,7)			
50		(20,9)	(12,8)			
51		(20,9)	(12,8)			
52		(20,9)	(12,8)			
53		(20,9)	(12,8)			

Number	(n, m) for SWNT	(n, m) for outer layer of DWNT	(n, m) for inner layer of DWNT	(n, m) for outer layer of TWNT	(n, m) for middle layer of TWNT	(n, m) for inner layer of TWNT
54		(18,13)	(14,7)			
55		(18,13)	(14,7)			
56		(18,10)	(13,6)			
57		(18,10)	(13,6)			
58		(18,10)	(13,6)			
59		(18,10)	(13,6)			
60		(18,10)	(13,6)			
61		(21,8)	(11,10)			
62		(20,9)	(12,8)			

Supporting Data 1 (separate file)

Detailed chiral indices identified for each wall from the CNTs at the length positions of 20 mm and 70 mm, including their chiral angles and semiconducting or metallic type.

References

[1] W. Wu, J. Yue, X. Lin, D. Li, F. Zhu, X. Yin, J. Zhu, J. Wang, J. Zhang, Y. Chen, X. Wang, T. Li, Y. He, X. Dai, P. Liu, Y. Wei, J. Wang, W. Zhang, Y. Huang, L. Fan, L. Zhang, Q. Li, S. Fan, K. Jiang, *Nano Res.* **2015**, *8*, 2721.

[2] Z. Zhu, N. Wei, W. Cheng, B. Shen, S. Sun, J. Gao, Q. Wen, R. Zhang, J. Xu, Y. Wang, F. Wei, *Nat. Commun.* **2019**, *10*, 4467.

[3] Z. Zhu, N. Wei, H. Xie, R. Zhang, Y. Bai, Q. Wang, C. Zhang, S. Wang, L. Peng, L. Dai, F. Wei, *Sci. Adv.* **2016**, *2*, e1601572.

[4] S. Paterson, T. Vogwill, A. Buckling, R. Benmayor, A. J. Spiers, N. R. Thomson, M. Quail, F. Smith, D. Walker, B. Libberton, A. Fenton, N. Hall, M. A. Brockhurst, *Nature* **2010**, *464*, 275.

[5] C. White, K. A. Selkoe, J. Watson, D. A. Siegel, D. C. Zacherl, R. J. Toonen, *Proc. Royal Soc. B* **2010**, *277*, 1685.

[6] K. Liu, J. Deslippe, F. Xiao, R. B. Capaz, X. Hong, S. Aloni, A. Zettl, W. Wang, X. Bai, S. G. Louie, E. Wang, F. Wang, *Nat. Nanotechnol.* **2012**, *7*, 325.

[7] T. Tomie, S. Inoue, Y. Matsumura, *Chem. Phys. Lett.* **2012**, *533*, 56.

[8] D. L. Plata, E. R. Meshot, C. M. Reddy, A. J. Hart, P. M. Gschwend, *ACS Nano* **2010**, *4*, 7185.
